# Demographic and genetic viability of a medium-sized ground-dwelling mammal in a fire prone, rapidly urbanizing landscape

**DOI:** 10.1371/journal.pone.0191190

**Published:** 2018-02-14

**Authors:** Cristina E. Ramalho, Kym M. Ottewell, Brian K. Chambers, Colin J. Yates, Barbara A. Wilson, Roberta Bencini, Geoff Barrett

**Affiliations:** 1 Western Australian Department of Biodiversity, Conservation and Attractions, Perth, Western Australia, Australia; 2 School of Biological Sciences, The University of Western Australia, Perth, Western Australia, Australia; 3 School of Agriculture and Environment, The University of Western Australia, Perth, Western Australia, Australia; Sichuan University, CHINA

## Abstract

The rapid and large-scale urbanization of peri-urban areas poses major and complex challenges for wildlife conservation. We used population viability analysis (PVA) to evaluate the influence of urban encroachment, fire, and fauna crossing structures, with and without accounting for inbreeding effects, on the metapopulation viability of a medium-sized ground-dwelling mammal, the southern brown bandicoot (*Isoodon obesulus*), in the rapidly expanding city of Perth, Australia. We surveyed two metapopulations over one and a half years, and parameterized the PVA models using largely field-collected data. The models revealed that spatial isolation imposed by housing and road encroachment has major impacts on *I*. *obesulus*. Although the species is known to persist in small metapopulations at moderate levels of habitat fragmentation, the models indicate that these populations become highly vulnerable to demographic decline, genetic deterioration, and local extinction under increasing habitat connectivity loss. Isolated metapopulations were also predicted to be highly sensitive to fire, with large-scale fires having greater negative impacts on population abundance than small-scale ones. To reduce the risk of decline and local extirpation of *I*. *obesulus* and other small- to medium-sized ground-dwelling mammals in urbanizing, fire prone landscapes, we recommend that remnant vegetation and vegetated, structurally-complex corridors between habitat patches be retained. Well-designed road underpasses can be effective to connect habitat patches and reduce the probability of inbreeding and genetic differentiation; however, adjustment of fire management practices to limit the size of unplanned fires and ensure the retention of long unburnt vegetation will also be required to ensure persistence. Our study supports the evidence that in rapidly urbanizing landscapes, a pro-active conservation approach is required that manages species at the metapopulation level and that prioritizes metapopulations and habitat with greater long-term probability of persistence and conservation capacity, respectively. This strategy may help us prevent future declines and local extirpations, and currently relatively common species from becoming rare.

## Introduction

Worldwide, urbanization is driving the rapid and large-scale clearing and fragmentation of natural ecosystems [1], with immediate and on-going consequences for mammalian wildlife in and nearby metropolitan regions (e.g., [2, 3]). While some species are highly sensitive to urbanization and are quickly lost at the onset of urban development, others are able to persist in small and relatively isolated remnant populations. These populations may appear stable, but are subject to demographic, genetic, and environmental stressors, as well as demographic and environmental stochasticity, which combine to accentuate their extinction risk, a process known as the small-population paradigm [4, 5].

Small- to medium-sized ground-dwelling mammals can be particularly vulnerable to fragmentation in urban areas for a number of reasons [6–8]. Firstly, loss of habitat connectivity due to housing and road encroachment impairs dispersal between habitat patches, diminishing habitat availability and preventing habitat recolonization after local extinction. Loss of connectivity can also reduce gene flow, which can lead to inbreeding depression and loss of genetic diversity, with consequent reduced fitness and reduced ability to adapt to environmental change [5]. Roads and other linear structures are a major cause of isolation and subsequent genetic differentiation, but also direct mortality through vehicle collision [9]. Secondly, the simplification of habitat and consequent reduction of dense vegetation cover—due to altered fire regimes, for example—reduces shelter and nesting habitat resources, which increase predation exposure, and can affect breeding and recruitment (e.g., [6]).

Wildlife road crossing structures (underpasses and overpasses) are increasingly used to mitigate the impacts of roads on wildlife and to restore habitat connectivity in urban areas (e.g., [10, 11]). Yet, there is a recognized lack of understanding about the effectiveness of these structures in restoring functional connectivity in impacted populations [12, 13]. Importantly, whether road crossings provide opportunity for gene flow to efficiently reduce inbreeding and genetic drift in isolated populations has seldom, if ever, been analysed [14–16].

In Mediterranean-climate cities, urbanization is associated with increased fire frequency, especially at intermediate human population densities, such as those found in peri-urban areas [17]. In these cities, preventing the decline and local extirpation of small- to medium-sized ground-dwelling mammal species requires an understanding of how habitat connectivity, small population processes (e.g., inbreeding depression), and fire regimes interact to affect metapopulation dynamics. Population viability analysis (PVA) provides a robust framework to address this challenge, allowing landscape managers to forecast and compare the relative impact of different stressors and management scenarios on population size and extinction risk [18, 19].

In this study, we used PVA to investigate the viability of two metapopulations of a medium-sized ground dwelling Australian marsupial inhabiting relatively small, fire-prone urban remnants connected by road underpasses. We compared the impacts of five scenarios, with and without accounting for inbreeding effects: (*i*) increased urban encroachment, (*ii*) removal of underpasses, (*iii*) increased number of underpasses, (*iv*) local- and (*v*) regional-scale fire. By modelling population dynamics using demographic and genetic data, we build on previous studies [11, 20], including a PVA for the same species [21], to elucidate how habitat connectivity, inbreeding depression, and fire regimes interact to affect the persistence of small metapopulations of medium-sized ground-dwelling mammals in urbanizing landscapes. Importantly, our study assesses how underpasses can mitigate the predicted demographic and genetic consequences of loss of habitat connectivity due to roads on wildlife populations.

## Material and methods

### Study species

The southern brown bandicoot (*Isoodon obesulus*, Shaw 1797) is an omnivorous, medium-sized ground-dwelling marsupial (0.4–2 kg, 28–33 cm; family Peramelidae). The species was once widespread across temperate southern Australia. However, habitat loss and fragmentation, predation by introduced carnivores, and changes to fire regimes have resulted in severe decline across most of its geographic range [22]. Nevertheless, the species is one of the very few ground-dwelling marsupials within the most extinction-prone 'critical weight range' group, i.e., 0.035–5.5 kg [23, 24], that persists in peri-urban areas of Australian state capital cities [21, 25]. The eastern subspecies *I*. *obesulus obesulus* is federally listed as endangered [26], whereas the western subspecies *I*. *obesulus fusciventer*, here studied, occurs in Perth and is state-listed as a priority species.

*Isoodon obesulus* inhabits dense, scrubby, often swampy vegetation adjacent to less dense areas (e.g., pastures, urban lawns, and post-fire regenerating heathland) that are used for foraging [27]. Home ranges vary between 0.5–5 ha, with smaller individuals and females typically occupying smaller areas than larger males [22]. The species has limited dispersal ability across cleared habitat, but is able to use vegetated corridors of either native or non-native species < 350 m to disperse between habitat patches [28]. Longer dispersal distances (up to 2 km) have also been recorded in forestry areas [29].

### Study area

Perth is a sprawling city with a development footprint that extends over 140 km along the coast, and a population of 2 million that is estimated to reach 3.2 million by 2030 [30]. Since the 1960s, urbanization has been the main driver of habitat fragmentation in the metropolitan region. Remnant vegetation persists in a few large conservation and Crown Land areas on the city boundaries, small and isolated urban reserves, roadside verges, and rural private properties [31].

**Fig 1:**
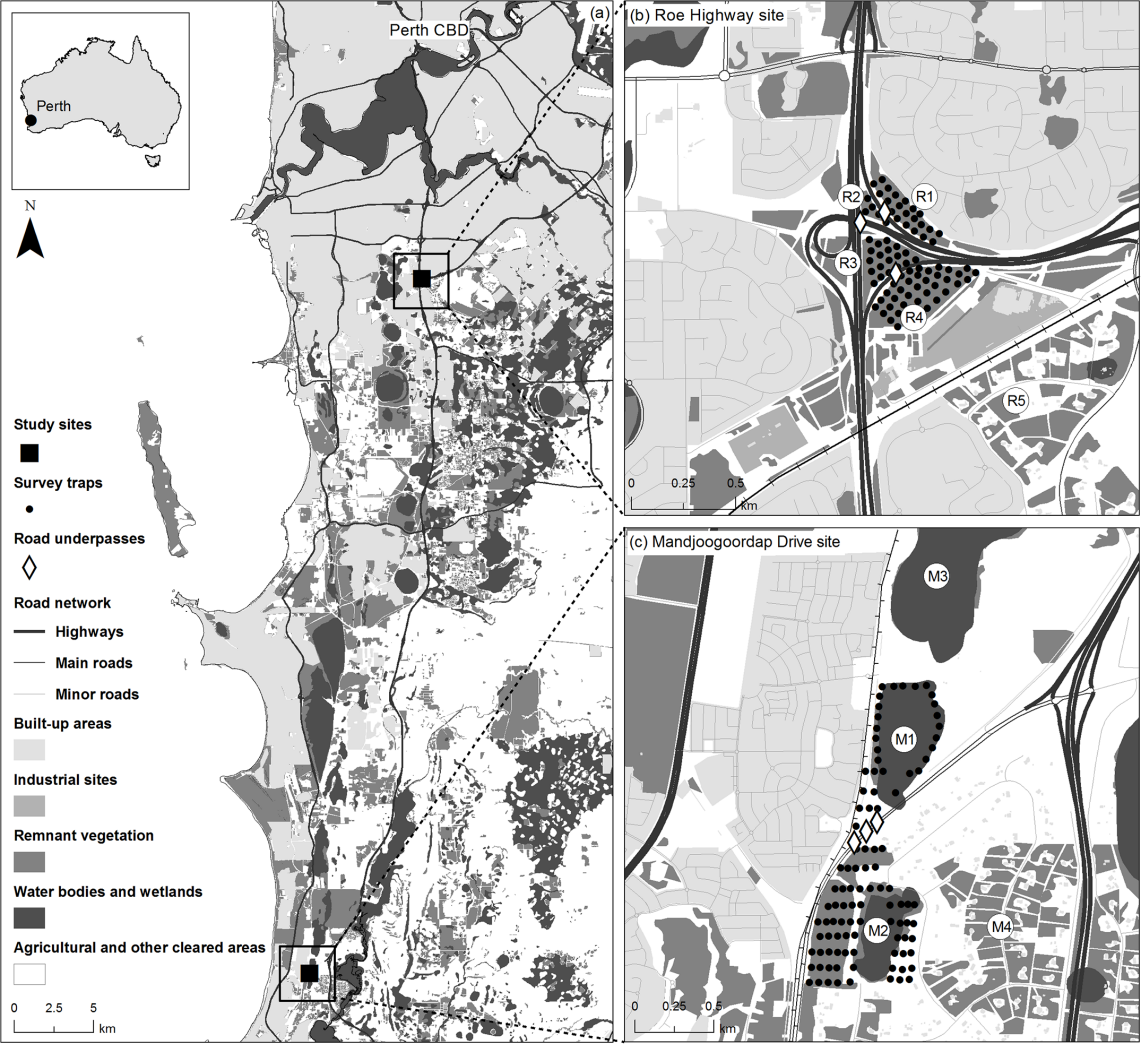
Study area in Perth, Western Australia. (a) Map of study area showing the location of the two study sites (b) Roe Highway and (c) Mandjoogoordap Drive, in Perth, Australia.

We surveyed two *I*. *obesulus* metapopulations inhabiting patches of remnant vegetation surrounding two different entrances to the Kwinana freeway, a main N-S oriented highway serving the Perth Metropolitan Area (Fig 1). The two sites, here named ‘Roe Highway’ (32°05’13.07”S, 115°51’06.11”E) and ‘Mandjoogoordap Drive' (32°28’51.28”S, 115°46’15.35”E), are isolated from each other and located 45 km apart. The Roe Highway site is composed of four patches of remnant vegetation (R1 = 5.6 ha, R2 = 1 ha, R3 = 4.6 ha, and R4 = 9.5 ha), whereas the Mandjoogoordap Drive site is composed of two patches (M1 = 13.5 ha and M2 = 6.3 ha) (Fig 1). At both sites, habitat patches are separated by highway entrances, which are fenced (1.8 m tall, 50 mm wide chainmesh, 0.5 m buried skirt fence) and have fauna underpasses underneath them. Roe Highway has three underpasses, two enclosed (22 and 29 m long) and one open (25 m), and Mandjoogoordap Drive has three enclosed underpasses (34, 41 and 47 m long). These wildlife underpasses were incorporated in the road design of the Roe Highway and Mandjoogoordap Drive, and were built in 2007 and 2009, respectively.

The Roe Highway site is highly isolated, being surrounded by the highway, suburbia, and an industrial site (Fig 1). The four patches are fully fenced, except when adjoining suburbia, where a mixture of suburban fencing occurs. Although very unlikely, dispersal of *I*. *obesulus* may occur with remnant habitat located southeast of the site (R5; edge-to-edge distance of 320 m; Fig 1). In contrast, the Mandjoogoordap Drive site is unfenced (except for the highway access) and bordered by a main road and suburbia to the west, and a rural landscape with remnant vegetation in the other directions. Dispersal is possible to and from two habitat patches, M3 and M4, located 310 m and 140 m from M1 and M2, respectively (Fig 1). Thus, despite being both relatively small metapopulations, landscape connectivity is greater at the Mandjoogoordap Drive site.

The vegetation at the study sites varies from open *Banksia* woodland with a relatively sparse shrub understorey in up-land areas (predominant at the Roe Highway site), to dense mid- and understorey heath surrounding swampy lowland areas (predominant at the Mandjoogoordap Drive site). Habitat quality at both sites is generally good and similar across the different surveyed patches. In terms of the surrounding habitat patches, M3 is broadly similar to M1 and M2. However, M4 and R5 have lower habitat suitability due to the partial clearing of the understorey, which is an important habitat component for *I*. *obesulus*.

### Trapping and monitoring of underpass use

Trapping sessions of four consecutive nights took place in the autumn, winter, and spring of 2012, and winter of 2013. Eighty-three trap sites were used at Roe Highway and 99 at Mandjoogoordap Drive, giving a total trapping effort of 1328 and 1584 trap nights at the two sites, respectively. Medium-sized wire cage traps (220 x 220 x 450 mm; Sheffield Wire Products, Welshpool WA) covered with a hessian sack and baited with universal bait (mixture of rolled oats, peanut butter and sardines) were set in a 50 m grid evenly spaced throughout the habitat patches (Fig 1). Traps were checked and cleared each morning at first light, before being reset. Trapped bandicoots were transferred to a dark cloth bag where they were weighed and standard measurements of pes length, head length, reproductive status and body weight were recorded. The sex of each bandicoot was also determined and their pouch (if female) was checked for pouch young. The number, sex, and crown-rump length of the pouch young were recorded. Tissue samples were taken from all adults (ear-biopsies), and stored in 100% ethanol for DNA analysis. A subset of adults (N = 37 at Roe Highway and N = 27 at Mandjoogoordap Drive) were genotyped at 12 microsatellite loci using conditions outlined in [32] to obtain allele frequencies and genetic diversity parameters for model input. Numbered ear tags and microchips (Trovan ID100, Trovan, Ltd., North Humberside, UK) implanted between the scapulae were used to individually identify animals. The use of the road underpasses was monitored using motion-activated cameras (Reconyx HC600) and flatbed microchip readers (Dorset ID ANT612/ LID650N), as described in [33].

All animal handling procedures were approved by The University of Western Australia’s Animal Ethics Committee (approval numbers RA/3/100/539, RA/3/100/1213 and RA/3/100/1117) under licenses provided by the Western Australian Department of Biodiversity, Conservation and Attractions (License numbers SF009026, SF009580, SF008789, SF008410, and SF008197). Animal handling procedures also followed the Australian Code of practice for the Care and Use of Animals for Scientific Purposes endorsed by the National Health and Medical Research Council of Australia [34].

### Population viability analysis

The viability of the two studied metapopulations was primarily analysed using the software RAMAS GIS 5.0 [35]. Estimates of demographic and life history parameters for *I*. *obesulus* were derived from field work, published literature, personal observations and, in some circumstances, assumed based on our best knowledge of the species (Table 1). We provide a description of the model below. Further details about model parameterization are in S1 Appendix.

**Table 1:** Demographic and population parameters used for the population viability analysis of *I*. *obesulus*, and respective data sources.

Parameter	Value		Data source
	Roe Highway	Mandjoogoordap Drive	
**Demographic rates**			
Age at first breeding	4 months		[22, 36, 37]
Maximum reproductive age	4 years		[22, 27]
Number of litters per year	2.61		[27]
% adult females breeding	100		Assumed*
% young adult males breeding	50		Assumed*
% older adult males breeding	100		Assumed*
Mean litter size at birth	2.65 (N = 18 litters)	2.65 (N = 25 litters)	Field data
Sex ratio at birth (M/F)	0.47/0.53	0.31/0.69	Field data
Fecundity (daughters per female)	0.998 ± 0.100	1.133 ± 0.113	Field data
Fecundity (sons per female)	0.885 ± 0.089	0.509 ± 0.051	Field data
Survival rates of juvenile females	0.384 ± 0.038	0.384 ± 0.038	Field data
Survival rates of adult females	0.817 ± 0.096	0.712 ± 0.118	Field data
Survival rates of juvenile males	0.384 ± 0.038	0.384 ± 0.038	Field data
Survival rates of young adult males	0.677 ± 0.192	0.706 ± 0.103	Field data
Survival rates of older adult males	0.677 ± 0.192	0.706 ± 0.103	Field data
**Population parameters**			
Maximum growth rate (*R*_*max*_)	1.31		Field data
Density dependence affects	Survival rates		Assumed
Density dependence is based on	All stages		[21]
Density dependence type	Scramble		Assumed
Temporal trend in *K*	0.033		[38]
Carrying capacity (*K*)	85 (R1 = 23 ± 2.3; R2 = 4 ± 0.4; R3 = 19 ± 1.9; R4 = 39 ± 3.9)	116 (M1 = 39 ± 3.9; M2 = 77 ± 7.7)	Field data
Initial abundances	37 (R1 = 23; R2 = 4; R3 = 3; R4 = 7)	27 (M1 = 9; M2 = 18)	Field data

(* *Based on* Ottewell and Chambers, personal observation)

*Isoodon obesulus* produces on average 2.61 litters in a year [27], typically in spring and winter, possibly due to the higher abundance of food resources available during this period. Gestation varies between 12–15 days and weaning between 60–70 days [22]. Sexual maturity is reached at 4–6 months [22, 36, 37]. Maximum recorded longevity is four years [22, 27].

We developed a stage and sex-structured model with four-month time steps to reflect the species biology and time of first reproduction. We included males and females separately in the model because the species has a polygynous and, to a lesser extent, polyandrous mating system (Ottewell and Chambers, personal observation; see also [39]). Paternity analysis shows that although all adult males can potentially breed, younger, smaller males are largely outcompeted by older, larger males and as a consequence have a lower breeding participation rate (Ottewell and Chambers, personal observation). Based on this information we constructed a matrix model with five stages: juvenile females, adult females, juvenile males, young adult males, and older adult males.

Fecundity rates were estimated using field-collected data from the two metapopulations and an additional three sites surveyed over the same period in the study area. Fecundity (the number of male/female offspring per year produced by a female) was calculated for a four-month time step using the formula [(survival rate of adult females * number of litters per year * mean litter size at birth * sex ratio (male/female)) ÷ 3]. Juvenile survival rates were calculated as the field-collected survival rate of pouch young multiplied by the survival rates of sub-adults estimated by [27]. Adult survival rates were estimated with field-collected mark-recapture data from the two metapopulations and the robust design model using the program MARK [40]. These rates were initially calculated for the time-periods between capture occasions, which were 3 months for the periods between the first 3 capture occasions, and 9 months for the final interval. These survival estimates were then converted to a four month time step by raising the three month estimates to the power of 4/3 (1.3333) and the nine month estimate to the power of 4/9 (0.4444).

Population densities were estimated using a spatially explicit capture-recapture model [41] and a maximum-likelihood estimator with the buffer distance set at 250 m, which was approximately four times the trap-revealed-range statistic [41]. Initial abundances were calculated by multiplying the estimated mean density in each patch over the survey period by its area. A stable stage distribution was assumed.

Ecological carrying capacity (*K*) for *I*. *obesulus* is poorly understood. Evidence of sustained high densities in areas that are predator-free and/or have additional food resources [42] is consistent with the patterns observed in the two study populations, and suggests that *K* is dependent on the landscape context. As such, for the Roe Highway metapopulation, we estimated *K* as the mean maximum density (bandicoots/ha) multiplied by patch size (*K* = 4.12*patch size). For the Mandjoogoordap Drive metapopulation, we used the formula (*K* = 2*patch size), with the value of 2 bandicoots/ha being the maximum population density observed by Thomas [36] and Chambers in nearby reserves (also see [21]).

We used a ‘scramble’ model (Ricker-type function) of density dependence, which assumes that resources are shared more or less equally among individuals, with a maximum growth rate (*R*_*max*_) of 1.31 per four month period. *R*_*max*_ is the maximal proportion by which the population increases at each time step when there are no density dependence effects [37], and was calculated based on the population growth observed between May 2012 and May 2013 at another site in the Perth Metropolitan Area where the species was rapidly recovering from low densities after fire (see S1 Appendix). Further, we assumed that density affects only survival rates (not fecundity), and depends on the abundance of individuals in all stages (as in [21]).

Dispersal rates between surveyed habitat patches were estimated as the proportion of micro-chipped animals that were recorded using the underpasses, whereas dispersal rates between surveyed and surrounding habitat patches were assumed based on the landscape permeability (S1 Appendix, Tables A and B). We assumed a relative dispersal value of 1 for juveniles and 0.2 for adults, given that dispersal occurs primarily in juveniles, with adults typically showing high site fidelity [27]. We included dispersal amongst surveyed and surrounding habitat patches in our models, but did not include populations in these surrounding patches (M3, M4, R5) in the calculation of the total metapopulation size. Initial abundances and carrying capacity for M3 were assumed to be proportionally the same as for its nearby surveyed metapopulation. Parameters for M4 and R5 were assumed to be 50% of their nearby surveyed metapopulations, given their lower habitat suitability (see S1 Appendix, Table C).

Demographic and environmental stochasticity were included in the models. Demographic stochasticity was modelled by sampling, after each time step, the number of survivors from a binomial distribution, and the number of offspring from a Poisson distribution [35]. Environmental stochasticity was modelled by randomly drawing values of fecundity and survival from lognormal distributions determined by their mean and standard deviation values. Standard deviations for survival rates and *K* were calculated from the data collected, whereas standard deviations for fecundity rates were assumed to be 10% of the estimated mean (see S1 Appendix for more details). All simulations were run for 50 years (150 time steps) and replicated 1000 times.

### Management scenarios

We modelled the effect of five management scenarios and their combinations on the viability of the two *I*. *obesulus* metapopulations: (i) increased urban encroachment, (ii) removal of underpasses, (iii) increased number of underpasses, (iv) local- and (v) regional-scale fire.

Firstly, we tested the effects of urban encroachment leading to a total loss of connectivity between the surveyed patches and surrounding remnant vegetation (dispersal rates = 0). Secondly, we tested the effects of road underpasses in two different scenarios: no underpasses (dispersal rates between surveyed patches = 0); and increased number/efficiency of underpasses. The latter was modelled by increasing the recorded dispersal rates by 2-fold for the Roe Highway model and 10-fold for the Mandjoogoordap Drive model. A greater relative increase of underpass dispersal rates in the Mandjoogoordap Drive model was used because the recorded rates at this site were very low, and a 10-fold increase equalled the average dispersal rate observed at the Roe Highway site (see S1 Appendix, Tables A and B). Thirdly, we investigated the effects of two types of fire spatial extent: local (probability of fire occurrence is independent for each habitat patch); and regional (fire affects all patches at once) [35]. Both types of fire are possible at the study sites because the habitat patches are relatively close together and the fuel loads relatively connected. Unplanned fires may be contained within individual patches if response times are swift and fire weather is not severe, but if response times are slow or fire weather severe then multiple patches may burn during the same fire. We assumed that fire decreases population abundances by 70% (based on the long-term study of the impacts of a wildfire on *I*. *obesulus* population dynamics by [43]) and *K* by 50%, and that *K* takes 5 years to recover to pre-fire levels at a 3.3% rate (based on vegetation density measures by [38]). Moreover, we estimated that the probability of a fire occurrence is 0.025 per time step (*i*.*e*., one fire occurrence every 13 years), based on a desktop GIS analysis of the fire history (1971–2013) in the region.

We compared the impacts of the different scenarios on expected minimum abundance (EMA), probability of decline, and extinction risk. We focused on the EMA results because they provide a more robust indication of propensity to decline than extinction risk [44].

### Inbreeding depression and genetic diversity

We used genotypic data from each metapopulation to investigate the impacts of inbreeding and genetic diversity loss on population viability. Because several of the assumptions required to satisfactorily model inbreeding depression in RAMAS GIS could not be met, we used the individual-based software Vortex 10.0.0.1 [45], which allows simultaneous tracking of demographic and genetic parameters over time. We replicated the demographic models in Vortex adding additional information on mating patterns. Although all adult males can potentially breed, younger, smaller males are largely outcompeted by older, larger males in gaining access to mates (Ottewell and Chambers, personal observation). We incorporated this information into estimates of reproductive rates and mate monopolization (S1 Appendix, Table D). We seeded each model with allele frequency data generated from 12 microsatellite loci genotyped in each metapopulation (S1 Table). In addition, we applied inbreeding depression to each of the base models and scenarios affecting dispersal (urban encroachment, underpasses) to assess the impact of inbreeding on population viability. Inbreeding depression was applied at two rates: (i) ‘mild’ 3.14 diploid lethal equivalents (rate estimated from captive populations; [46]); and (ii) ‘stressful’ 6.29 diploid lethal equivalents (estimated from wild populations; [47]), representing a reduction in first-year survival of inbred individuals due to recessive alleles. We assessed the impacts of these genetic effects on EMA, allelic diversity, and expected and observed heterozygosity. In addition, we estimated genetic differentiation (G_ST_) between the surveyed habitat patches at each site at the end of the simulation (50 years). The Vortex and RAMAS models produced largely congruent projections of metapopulation abundance and extinction risk under the different management scenarios, with minor variation in EMA (S1 Fig).

### Sensitivity analysis

To assess the RAMAS models’ sensitivity to variation or uncertainty in parameter estimates, we analysed the effects on EMA of changes (ranging from -10% to +10%) in mean demographic rates, environmental stochasticity (SD of demographic rates), *K*, and *R*_*max*_.

## Results

**Fig 2:**
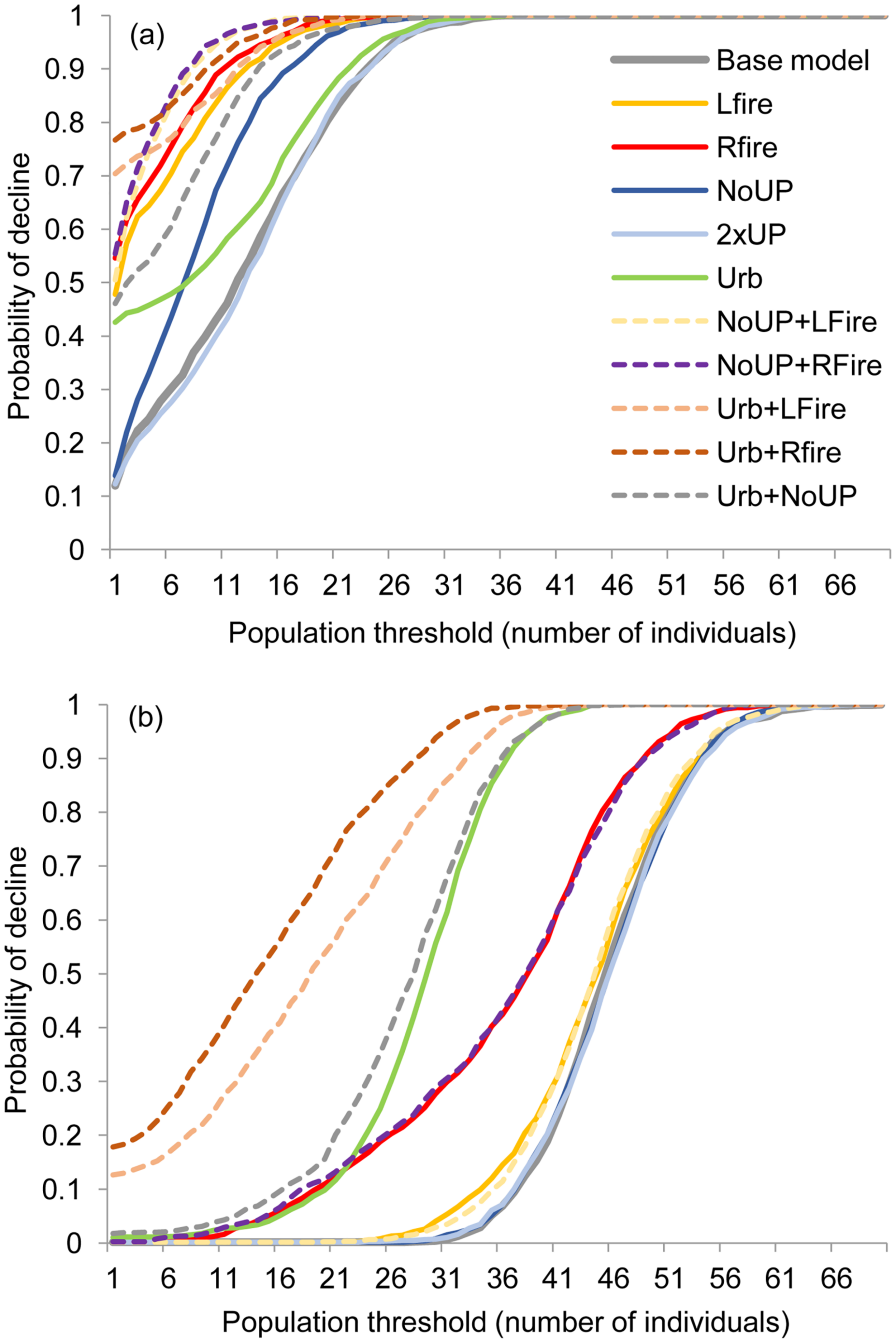
Risk curves for the base model and the alternative management scenarios estimating the probability of the Roe Highway (a) and Mandjoogoordap Drive (b) metapopulations falling below a specified population threshold (x-axis) during the next 50 years. Scenarios increasing the number of underpasses and those combining three management options are not represented for visual simplification and because the former had negligible influence on population viability.

Initial abundances in Roe Highway and Mandjoogoordap Drive metapopulations were 37 and 27 individuals, respectively. The base model (if current conditions were to be maintained) for the Roe Highway metapopulation projected a small increase in the average population abundance up to 56 individuals (S2A Fig) and an extinction risk of 12% in the next 50 years. This extinction risk increased up to 76% in the worst-case scenario combining the effects of a widespread fire with loss of habitat connectivity, both among the surveyed patches and with the surrounding landscape (Table 2, Fig 2). In contrast, the base model for the Mandjoogoordap Drive metapopulation projected a substantial increase up to 180 individuals (S2A Fig) and a null extinction risk. However, in the worst-case scenario, a 20% extinction risk was predicted for this metapopulation (Table 2, Fig 2).

**Table 2:** Expected minimum abundance (EMA) and probability of a 25%, 50%, 75%, and 100% decline in metapopulation size at least once in the next 50 years, for the two studied *I*. *obesulus* metapopulations, under the alternative management scenarios.

Scenarios	Roe Highway					Mandjoogoordap Drive				
	25%	50%	75%	100%	EMA	25%	50%	75%	100%	EMA
**Base model**	0.98	0.79	0.43	0.12	11.5	0.00	0.00	0.00	0.00	45.0
**Local fire (LFire)**	1.00	0.98	0.84	0.48	3.8	0.00	0.00	0.00	0.00	43.6
**Regional fire (RFire)**	1.00	0.99	0.89	0.55	3.1	0.12	0.05	0.01	0.00	35.4
**No underpasses (NoUP)**	1.00	0.95	0.67	0.14	7.4	0.00	0.00	0.00	0.00	45.0
**Double underpasses (2xUP)**	0.98	0.79	0.40	0.12	11.8	0.00	0.00	0.00	0.00	45.1
**Urban encroachment (Urb)**	0.99	0.84	0.55	0.43	8.6	0.12	0.04	0.02	0.01	28.0
**Lfire+NoUP**	1.00	1.00	0.95	0.51	2.3	0.00	0.00	0.00	0.00	43.6
**Lfire+2xUP**	1.00	0.99	0.84	0.50	3.7	0.00	0.00	0.00	0.00	43.5
**Rfire+NoUP**	1.00	1.00	0.95	0.55	2.1	0.13	0.05	0.01	0.00	35.3
**Rfire+2xUP**	1.00	0.98	0.88	0.60	2.8	0.13	0.06	0.01	0.00	35.5
**Lfire+Urb**	1.00	0.99	0.85	0.70	2.8	0.56	0.37	0.21	0.13	17.8
**Rfire+Urb**	1.00	1.00	0.92	0.77	2.0	0.73	0.52	0.32	0.18	13.7
**Urb+NoUP**	1.00	0.97	0.77	0.46	4.9	0.08	0.03	0.03	0.02	26.3
**Urb+2xUP**	0.99	0.83	0.54	0.42	8.9	0.08	0.02	0.01	0.01	29.3
**Lfire+NoUP+Urb**	1.00	1.00	0.96	0.77	1.3	0.76	0.53	0.32	0.18	13.5
**Rfire+NoUP+Urb**	1.00	1.00	0.97	0.76	1.4	0.79	0.58	0.32	0.20	12.3

**Fig 3:**
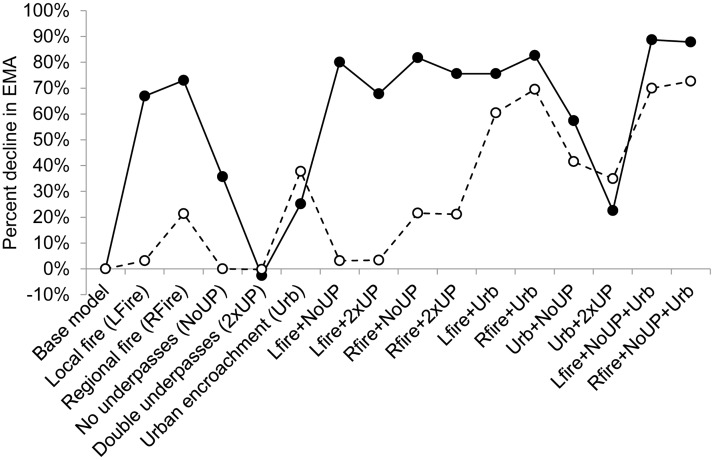
Projected declines in expected minimum abundance (EMA) under alternative management scenarios in relation to the base model for the Roe Highway (solid line) and Mandjoogoordap Drive (dashed line) metapopulations.

Fire occurrence was projected to have a substantial negative impact on the viability of the more isolated Roe Highway metapopulation, with a 67–73% decline in EMA compared to the base model, and a 48–55% extinction risk, in the case of a local and regional fire, respectively (Table 2, Figs 2 and 3). The combined effects of fire and further isolation through loss of underpasses or urban encroachment decreased EMA by 80–82% and 76–83%, respectively, practically driving the metapopulation to local extinction (Table 2, Figs 2 and 3).

Urban encroachment had a marked negative impact on the viability of the Mandjoogoordap Drive (38% decline in EMA), and to a lesser extent, Roe Highway (25% decline in EMA) metapopulations (Table 2, Figs 2 and 3). Importantly, under the current urban extent, the Mandjoogoordap Drive metapopulation was barely affected by a local fire, suffering a 21% decline in EMA in the case of a regional fire. However, in a scenario of urban encroachment, EMA declined by 60–70% and extinction risk increased by 13–18% (Table 2, Figs 2 and 3).

The underpasses currently in place at the Roe Highway site are important to the viability of this metapopulation and their removal would decrease EMA by 36% and increase the impacts of fire and urbanization (Table 2, Figs 2 and 3). In contrast, the removal of the underpasses at the Mandjoogoordap Drive site had little influence in the metapopulation's viability, even when taking fire or urbanization into account. Doubling the number of underpasses had only a marginal positive effect, if any, on both metapopulations (Table 2, Fig 3).

### Genetic diversity and inbreeding

**Fig 4:**
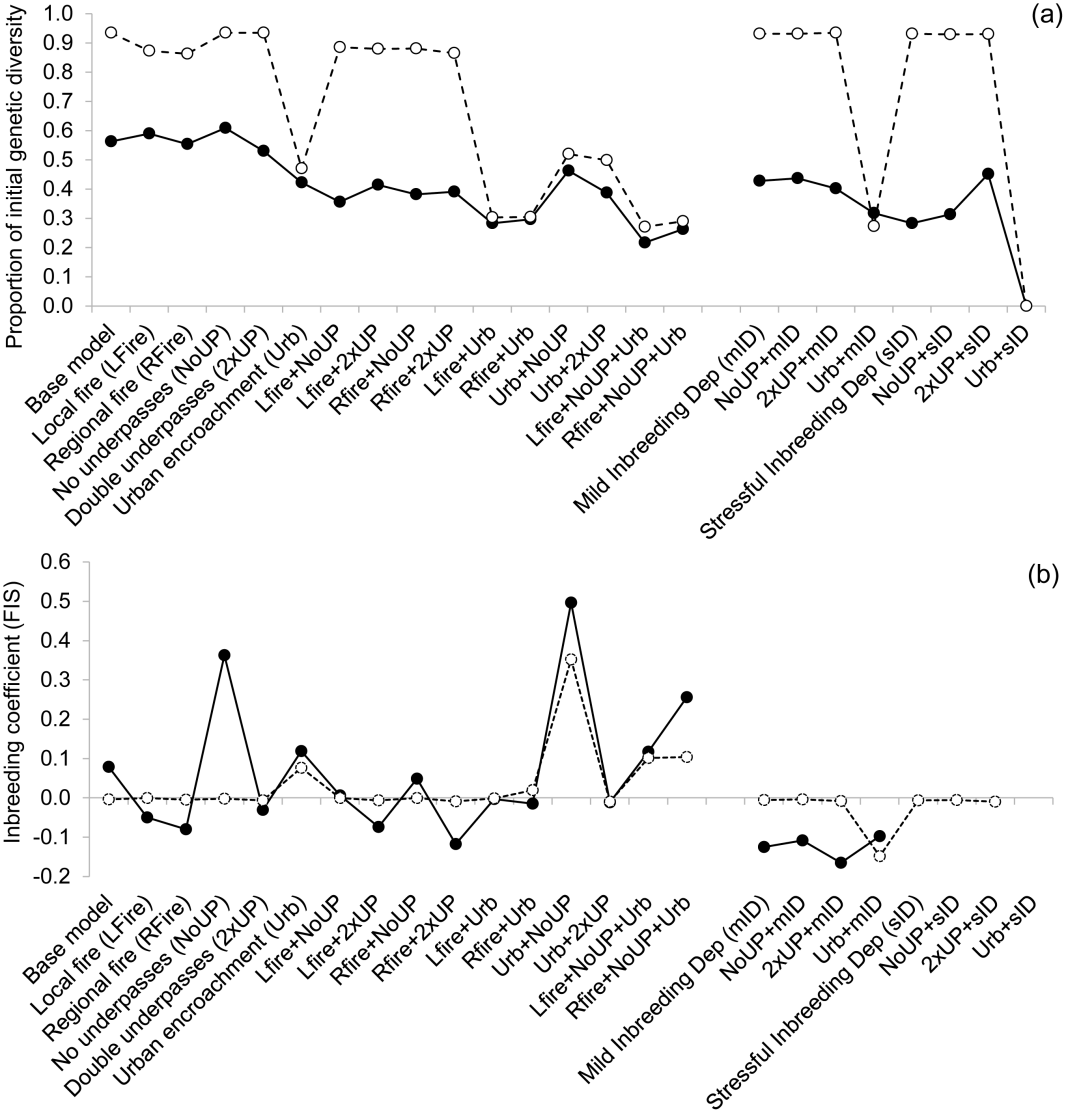
(a) Proportion of initial genetic diversity (expected heterozygosity) remaining, and (b) Wright’s inbreeding coefficient (F_IS_) under the alternative management scenarios after 50 years, for the Roe Highway (solid lines) and Mandjoogoordap Drive (dashed lines) metapopulations. F_IS_ was not calculated for scenarios with high extinction probabilities due to small sample size.

Both metapopulations had moderate genetic variation (H_E_ = 0.68 and 0.66; Roe Highway and Mandjoogoordap Drive, respectively) and no evidence of current inbreeding (F_IS_ = -0.013 and -0.017, respectively) (S1 Table). If current environmental conditions prevail, the Mandjoogoordap Drive metapopulation is predicted to be only slightly affected by inbreeding effects in 50 years (4–9% decline in EMA with mild and stressful inbreeding, respectively), whereas the Roe Highway metapopulation is predicted to be severely impacted (74–100% decline in EMA). Similarly, genetic diversity loss is predicted to be substantial for the Roe Highway metapopulation (56% of the starting heterozygosity expected to be remaining) but only minimal (94% remaining) for the Mandjoogoordap Drive metapopulation (Fig 4a).

The Roe Highway metapopulation was sensitive to the combined effects of fire and underpass removal (36–38% of the starting heterozygosity expected to be remaining), and both metapopulations were affected by urban encroachment, particularly when in combination with additional stressors (22–29% remaining) and stressful inbreeding, leading to complete extinction (Fig 4a). Removal of underpasses led to a marked accumulation of inbreeding and high genetic differentiation amongst the surveyed populations in the Roe Highway metapopulation, and in both metapopulations when urban encroachment led to further loss of habitat connectivity (Fig 4b, S2B and S2C Fig).

Under inbreeding depression, neither loss nor addition of underpasses greatly altered any of the metapopulation trajectories. However, urban encroachment with inbreeding depression led to a 90/97-100% decline in EMA in both metapopulations (S2B Fig).

### Sensitivity analysis

**Fig 5:**
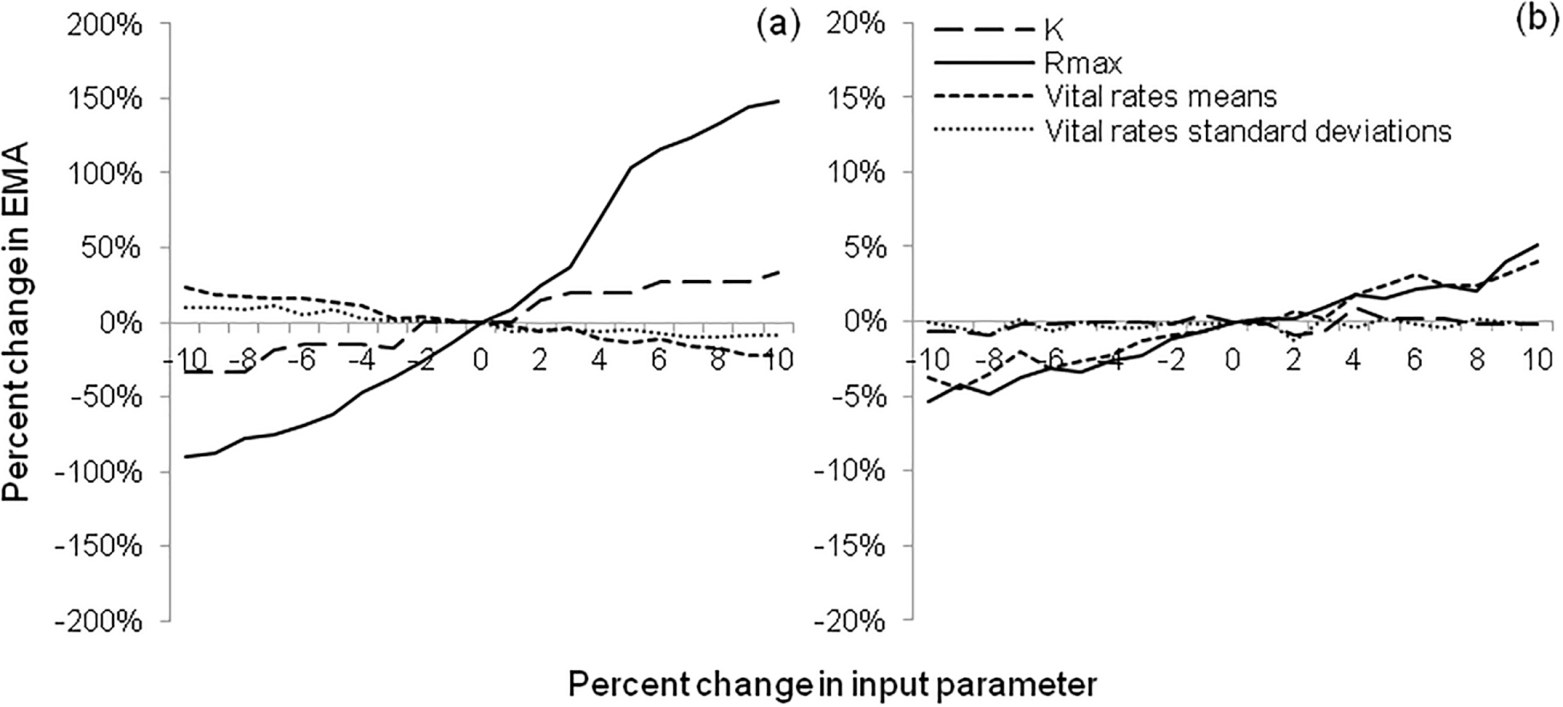
Sensitivity of population viability to changes in carrying capacity (*K*), maximum growth rate (*Rmax*), demographic rates (vital rates means), and environmental stochasticity (vital rates standard deviations) for the Roe Highway (a) and Mandjoogoordap Drive (b) metapopulations. Sensitivity is represented as the percent change in expected minimum abundance relative to the base model. Note that y-axes have different scales.

The Roe Highway metapopulation was substantially more sensitive to changes in the tested parameters than the Mandjoogoordap Drive metapopulation. Varying *R*_*max*_ had the greatest effect on both metapopulations, with a 10% decline in this parameter decreasing the EMA of Roe Highway and Mandjoogoordap Drive metapopulations by 90% and 5%, respectively (Fig 5). A 10% increase in *R*_*max*_ was predicted to increase the former’s EMA by 149%. Small changes in *K*, fecundity and survival rates also had a significant impact on the Roe Highway metapopulation, with a 10% reduction in *K* leading to a 100% decline in EMA (and vice-versa), and a 10% decline in fecundity and survival increasing its EMA by 24% and 10% respectively (Fig 5).

## Discussion

### Vulnerability to habitat connectivity loss and other interacting stressors

Housing and road encroachment leading to loss of habitat area and connectivity are major factors influencing the viability of *I*. *obesulus* metapopulations in rapidly urbanizing landscapes. Our study suggests that small *I*. *obesulus* metapopulations may be viable when embedded in relatively permeable and connected peri-urban landscapes, but become highly vulnerable to threatening factors, such as fire, once urbanization leads to their spatial isolation. Small and highly isolated *I*. *obesulus* metapopulations have an intrinsic high risk of population decline, genetic diversity loss, inbreeding depression, and local extinction in the long term. They are also more vulnerable to demographic and environmental stochasticity, as shown in the greater sensitivity of the Roe Highway metapopulation to variations in the demographic and environmental parameters (see also [48]). This metapopulation was particularly sensitive to changes in maximum growth rate, with lower growth rates reducing population viability, as has also been observed for the mountain pygmy possum [49]. Despite the striking difference between the two metapopulations in their sensitivity to this parameter, the estimate used is the best available and likely realistic, being based on field-collected, post-fire recovery data.

Fire substantially amplifies the effects of habitat fragmentation on *I*. *obesulus* metapopulations. It can lead to local extinction in small and isolated patches, as also observed by [50]. Furthermore, widespread, but also frequent or intense fires [51], eliminate long-unburnt vegetation patches, reducing habitat heterogeneity and complexity, which is a key feature for bandicoots and other fire-sensitive small and medium-sized ground-dwelling mammals [43, 52, 53]. These species depend on long-unburnt understorey for shelter and nesting [28, 43], also exploiting adjacent open habitat, including recently burnt areas, for food resources [54, 55]. The elimination of this habitat layer is associated with increased predation pressure and loss of nesting habitat, with consequent population decline [56], slow post-fire recovery [43], and/or reduced breeding and recruitment [52].

Although predation is a recognized threat for *I*. *obesulus* [21, 57, 58], we did not model its effects because of difficulty in doing so in a meaningful manner. Introduced predators are known to occur at both study sites and were recorded using the underpasses, including at the Roe Highway site, where the metapopulation was nearly at carrying capacity. This is despite the fact that fox predation following road construction had previously led to the extirpation of one the populations at this site [59], which may also explain the low population numbers recorded at the Mandjoogoordap Drive site (which was built two years later). The ability to sustain population sizes at high densities in the presence of introduced predators, as observed at the Roe Highway site, may indicate that the availability of anthropogenic resource subsidies in the peri-urban matrix may be leading to a reduction in predation pressure (the 'predation paradox'; [60, 61]). Also, *I*. *obesulus* abundance data from predator-managed peri-urban reserves were not available for our study region.

Our study showed that genetic changes, such as inbreeding, loss of genetic diversity, and population genetic differentiation, may go undetected in seemingly demographically stable populations, yet may contribute substantially to cumulative extinction risks [62, 63]. The observed accumulation of inbreeding when populations experienced isolation reinforces the importance of maintaining gene flow to avoid both the fitness decline and loss of evolutionary potential that is expected to occur with inbreeding and genetic drift, although some small populations may purge genetic load over the longer term to retain fitness [5]. The lack of inbreeding observed in the current populations (F_IS_ ≈ -0.01) may reflect a time lag effect, in that these populations were only fenced recently (~6 years when the study was conducted) and insufficient generations have passed to observe an effect of accumulated inbreeding. The polygamous mating system of *I*. *obesulus* may also buffer small populations from accumulation of inbreeding, at least in the short term. Nonetheless, meta-analyses have shown that the loss of genetic diversity at neutral genetic markers (as we have measured here) is often accompanied by an equivalent or greater loss of diversity at other highly variable adaptive genetic loci, such as major histo-compatibility complex (MHC) genes that are important in disease resistance [64, 65]. This is potentially an additional cause of decline for small, genetically-compromised populations, which are already likely to be susceptible to increased disease and parasite risk that typically accompanies urbanisation and exposure to domestic animals (e.g., [66]).

*I*. *obesulus* has several traits that influence its ability to persist and even increase in abundance in peri-urban areas with high native vegetation cover. These include: 1) high reproductive and growth rates [22, 37]; 2) ability to exploit the urban environment for food, water, and shelter [25, 67–69]; 3) omnivory, in opposition to species with a specialized diet, which tend to be negatively affected by urbanization [70]: and 4) the ability to use small and overlapping home ranges when resources are abundant [42]. These are traits typical of “urban adapter” species [1] and enable *I*. *obesulus* and other species (e.g., [71]) to take advantage of the increased resource availability that characterize urban and peri-urban areas compared to rural and natural areas [60, 61].

These traits, however, are seemingly insufficient to counteract the sensitivity of *I*. *obesulus* to habitat fragmentation. Habitat connectivity has been identified as the main predictor of occupancy of urban remnants for several small to medium-sized ground-dwelling mammals that, as *I*. *obesulus*, have low-to-moderate vagility across human-modified environments and specific habitat requirements [7, 8, 72]. These mammals are likely more vulnerable to urbanization than those that are highly vagile and perceive the urban matrix as relatively permeable, as well as those on the other side of the spectrum that have very limited dispersal ability and small home ranges, and are able to persist in isolated patches [7, 8]. Thus, while still relatively common, *I*. *obesulus* is likely to be in a trajectory of decline in Perth, given the ongoing habitat clearing, urban growth, and infill rates. This is consistent with the fact that the species is locally extinct in the more urbanized and older suburbs of Perth, even in relatively large remnants (400–450 ha; [25]), and is much less abundant in more urbanized cities (e.g., [21]).

### The mitigating role of road underpasses

Consistent with other studies [10, 11], our results showed that underpasses mitigate the impacts of roads on *I*. *obesulus* metapopulation viability by connecting habitat patches that would be otherwise not large enough to support viable populations, thus increasing habitat availability, and reducing the probability of inbreeding and genetic differentiation on metapopulations affected by increased urbanization. Three aspects should be highlighted. First, even with the current underpasses, the Roe Highway metapopulation was predicted to be affected by inbreeding depression and lose nearly half of its genetic diversity within 50 years because of sustained small population size and high spatial isolation. These results show how PVA using genetic data offers a tool to prioritize locations to place road crossing structures [15]. Second, the fact that underpasses had only a marginal influence on the viability of the Mandjoogoordap Drive metapopulation is likely to indicate an inadequate underpass design [73]. Underpasses at this site are relatively long, and long underpasses have been found to be used less by *I*. *obesulus* and other bandicoot species [33, 74]. Third, our models assumed that dispersers successfully established and bred in their recipient populations, contributing to effective gene flow amongst patches. While we do not have field data on the fate of dispersing individuals in our study, we have evidence from paternity analyses indicating successful reproduction in recipient populations for a limited number of animals using underpasses (Ottewell and Chambers, personal observation). However, since dispersal does not always result in gene flow [15, 16], further study is required to better parameterise the role of underpasses in providing functional connectivity. Consequently, our current estimates of dispersal via underpasses in these populations may be taken as the maximum potential rate of functional dispersal, while the realised rate may be lower (which could further exacerbate the genetic effects in these populations).

### Implications for conservation planning and ecosystem management

The fact that peri-urban areas are highly dynamic landscapes, from a spatial and temporal perspective [75–77], poses a major challenge for biodiversity conservation. In such environments, ‘what you see is not what you get’ [78], as the impacts of recent and ongoing habitat fragmentation and land-use change on biodiversity may take years to materialize [79], depending on the rate and spatial configuration of land-use change [76]. Setting conservation objectives and priorities in these ‘shifting’ landscapes is particularly challenging in the case of species that are vulnerable to habitat fragmentation but have some 'urban adapter' traits that allow them to tolerate or even respond positively to moderate levels of urbanization. These species may be perceived as common but be, in fact, on a trajectory of decline [80].

Given knowledge of the species’ habitat requirements, and based on the results of this study, we propose the following key interventions to safeguard the persistence of *I*. *obesulus* in urban remnants. These interventions are likely to benefit other small-to-medium sized vertebrates in the study area, and more broadly, inform conservation planning strategies to protect small-to-medium sized ground dwelling mammals in fire-prone, rapidly urbanizing landscapes.

1. Retention of remnant vegetation, since smaller populations are more vulnerable to multiple and interacting stressors and connectivity plays a major role in population viability.
2. Enhancement of existing dispersal corridors, and implementation of new corridors, especially in new residential developments. Dense, weedy areas can be used for dispersal and shelter by *I*. *obesulus* and other ground-dwelling vertebrates in modified landscapes [55, 81]. This demands thoughtful spatial and temporal planning in weed management and restoration practices, so that critical habitat is not removed before appropriate (structurally complex and dense) habitat is available.
3. Installment of road underpasses that enable dispersal and frequent use. Underpass design is a key factor determining their efficiency, and for *I*. *obesulus* they should be as short as possible and provide dense vegetation cover at the approaches. In the case of wide roads, a vegetated area (median strip) connecting two short underpasses is a better alternative to a single, long underpass [33].
4. Fire management that 1) limits the size of unplanned fires, and 2) promotes mosaic burning with the retention of significant areas of long-unburnt vegetation within the metapopulation’s range. This could be achieved by limiting burning to only one inter-connected patch at any of the sites, in any 5 year period. Mosaic burning has been shown to be important for the conservation of other taxa in fire-prone ecosystems [82].
5. Reintroduction of *I*. *obesulus* into areas where local extirpations have occurred but that still have reasonable habitat quality, size, and connectivity. Reintroductions into isolated patches are unlikely to be successful unless patches are controlled for predators and populations managed to minimize inbreeding.
6. Adoption of a triage approach [83] that prioritizes management efforts on metapopulations with greater long-term probability of persistence, and on habitat patches with greater long-term conservation capacity.

## Conclusions

The urbanization of peri-urban areas poses a major challenge for wildlife conservation. Small- to medium-sized ground-dwelling mammals that are sensitive to loss of habitat area and connectivity may persist in urban and peri-urban areas with moderate levels of habitat fragmentation, but are likely to decline under increasing urbanization. A proactive conservation approach that manages species at the metapopulation level and that prioritizes metapopulations with greater long-term probability of persistence is required to prevent future declines and local extirpations, and importantly, prevent relatively common species from becoming rare.

## Supporting information

S1 Appendix Extended information on the metapopulation model parameterization. (DOCX)

S1 Table Allele frequency and genetic diversity in the study metapopulations. (DOCX)

S1 Fig Expected minimum abundance under different management scenarios estimated using RAMAS and Vortex software. (DOCX)

S2 Fig Additional results. (DOCX)

S1 File Trapping data. (XLSX)
