# Supplementary material for: Demographic and genetic viability of a medium-sized ground-dwelling mammal in a fire prone, rapidly urbanizing landscape
Source: PLoS One. 2018 Feb 14;13(2):e0191190. doi: 10.1371/journal.pone.0191190 (PMC5812552; doi:10.1371/journal.pone.0191190)
Supplement: S1 Appendix — (DOCX) [file pone.0191190.s001.docx]

# S1 Appendix: Extended information on the parameterization of the *Isoodon obesulus* metapopulation model

The metapopulation viability of *Isoodon obesulus* in the two study sites located in the Perth Metropolitan Area, Western Australia, was analysed using the software RAMAS GIS 5.0 ([1](#_ENREF_1)). Because some of the assumptions required to satisfactorily model inbreeding depression in RAMAS GIS could not be met, we used the software Vortex v10.0.0.1 ([2](#_ENREF_2)) to analyse the impacts of inbreeding depression and genetic diversity loss on population viability.


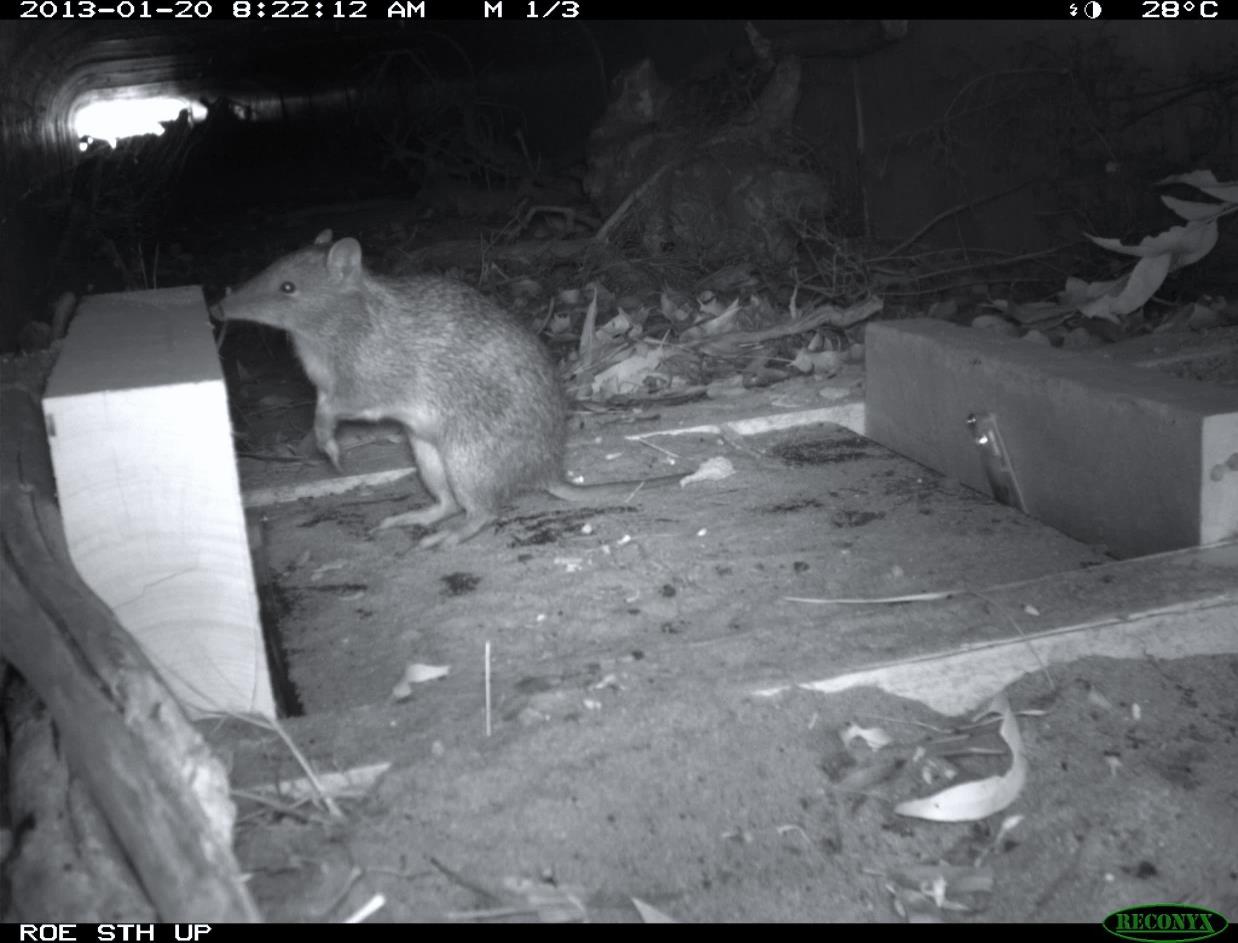

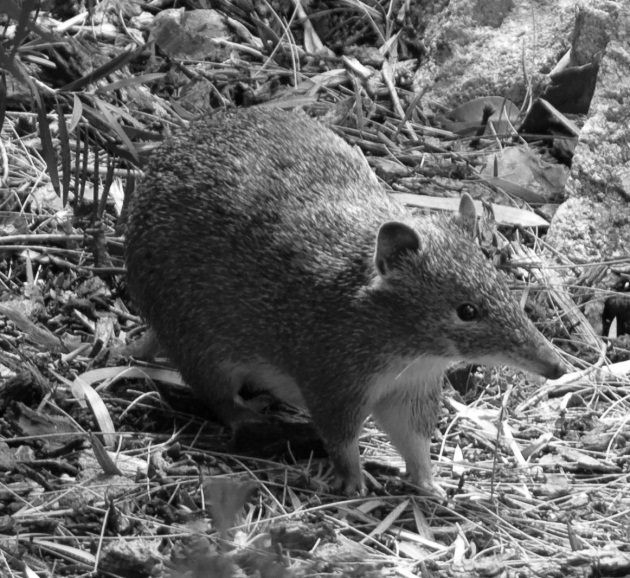


(b)

(a)

**Fig A.** The southern brown bandicoot (*Isoodon obesulus*) (a) using a wildlife underpass (photo taken with a Reconyx camera) and (b) in its habitat in remnant *Banksia* woodlands, in the Perth Metropolitan Area, Western Australia. Photographic credits: (b) Cristina E. Ramalho.

## Parameterization using RAMAS GIS

**Density dependence**

We used a maximum growth rate (*R_max_*) of 1.31 per four month period, which was calculated based on the population growth observed between May 2012 and May 2013 at another site in the Perth Metropolitan Area where the species was rapidly recovering from low densities after fire. Other maximum growth rates available in the literature for *I. obesulus* include annual values of 1.5, assumed by ([3](#_ENREF_3)), and 2.02, estimated by ([4](#_ENREF_4)) based on the age of first reproduction and annual fecundity.

**Stochasticity**

Environmental stochasticity was modelled by drawing randomly values of fecundity and survival from lognormal distributions determined by their mean and standard deviation values. Values were drawn from lognormal distributions to [reduce potential truncations due to high vital rates in some stages](#_ENREF_1) ([1](#_ENREF_1)). Constraints were imposed to ensure that all survival rates were within the bounds of 0 and 1. The effects of stochasticity on fecundity, survival, and carrying capacity were assumed to be correlated within the population.

**Dispersal**

As explained in the manuscript, dispersal rates between surveyed habitat patches were estimated as the proportion of micro-chipped animals that were recorded using the underpasses, whereas dispersal rates between surveyed and surrounding habitat patches were assumed based on the landscape permeability.

**Table A**. Dispersal rates for the Roe Highway metapopulation. Dispersal rates between surveyed habitat patches are in bold, while those between surveyed and surrounding habitat patches are in italic.

|  | R1 | R2 | R3 | R4 | *R5* |
| --- | --- | --- | --- | --- | --- |
| R1 |  | **0.15** | 0 | 0 | 0 |
| R2 | **0.15** |  | **0.05** | 0 | 0 |
| R3 | 0 | **0.05** |  | **0.1** | 0 |
| R4 | 0 | 0 | **0.1** |  | *0.01* |
| *R5* | 0 | 0 | 0 | *0.01* |  |

**Table B.** Dispersal rates for the Mandjoogoordap Drive metapopulation. Dispersal rates between surveyed habitat patches are in bold, while those between surveyed and surrounding habitat patches are in italic.

|  | M1 | M2 | *M3* | *M4* |
| --- | --- | --- | --- | --- |
| M1 |  | **0.01** | *0.05* | 0 |
| M2 | **0.01** |  | 0 | *0.05* |
| *M3* | *0.05* | 0 |  | 0 |
| *M4* | 0 | *0.05* | 0 |  |

Initial abundances and carrying capacity for M3 were assumed to be proportionally the same as for its nearby surveyed metapopulation. Parameters for M4 and R5 were assumed to be 50% of their nearby surveyed metapopulations, given their lower habitat suitability.

**Table C.** Patch size, estimated *K* and initial abundances of *I. obesulus* for the habitat patches surrounding the studied metapopulations.

| Parameter | R5 | M3 | M4 |
| --- | --- | --- | --- |
| Patch area (ha) | 27.3 | 822.2 | 90.8 |
| Carrying capacity | 56 ± 5.6 | 1644 ± 164.4 | 91 ± 9.1 |
| Initial abundances | 10 | 395 | 22 |

## Parameterization using Vortex

**Inbreeding depression and genetic diversity**

Inbreeding depression and genetic diversity were modelled using the software Vortex v10.0.0.1 ([2](#_ENREF_2)), an individual-based modelling environment able to incorporate genetic information. Stage-based transition rates developed in RAMAS GIS were converted to individual probabilities of survival, fecundity and dispersal in Vortex (Table D).

**Table D.** Demographic and life history parameters used in the Vortex population viability model of *I. obesulus* in the two study sites located in the Perth Metropolitan Area, Australia. All parameters were obtained from survey data, except when they were assumed (+) or obtained from the literature (*).

|  | **Roe Highway** |  | **Mandjoogoordap Drive** |
| --- | --- | --- | --- |
| **Scenario settings** |  |  |  |
| Number of iterations |  | 1000 |  |
| Number of years modelled | 50 (150 time steps) | | |
| Duration of each time step in days |  | 120 days |  |
| Number of populations | 4 |  | 2 |
| Extinction definition |  | Total N <1 |  |
| Order of events |  | Default |  |
| **Species Description** |  |  |  |
| Inbreeding depression |  |  |  |
| - Lethal equivalents | 3.14*^1^ / 6.29*^2^ | | |
| - % due to recessive lethal alleles |  | 50 ^+^ |  |
| EV correlation between reproduction and survival |  | 0.5 ^+^ |  |
| EV correlation among populations |  | 0.5 ^+^ |  |
| **Dispersal** |  |  |  |
| Age range | 4 months to 4 years | | |
| Dispersing sexes |  | Both |  |
| % survival of dispersers |  | 100 |  |
| Dispersal modifier (relative dispersal rate for juveniles (1) and adults (0.2)^+^) | = IF (A=1; (D*1);(D*0.2)) | | |
| Dispersal matrix | Table C |  | Table D |
| **Reproductive system** |  |  |  |
| Mating system |  | Polygynous |  |
| Age of first offspring (M + F) |  | 8 months |  |
| Maximum age of reproduction (M + F) |  | 4 years |  |
| Max number of broods/year |  | 1 |  |
| Max number of progeny/brood |  | 4 |  |
| Sex ratio at birth (M/F) | 0.47/0.53 |  | 0.31/0.69 |
| Number of litters per time step |  | 1 (2.61/year^*3^) |  |
| Density dependent reproduction |  |  |  |
| - % breeding at low density, P(0) |  | 90 |  |
| - % breeding at carrying capacity, P(K) |  | 45 |  |
| - Allee parameter, A |  | 0 |  |
| - Steepness parameter, B |  | 4 |  |
| **Reproductive rates** |  |  |  |
| % adult females breeding | Density-dependent function as above | | |
| - SD in % breeding due to EV |  | 10 |  |
| Distribution of broods, 1 brood |  | 100% |  |
| No. of offspring per female per brood |  | 2.65 ± 0.72 |  |
| **Mortality rates** |  |  |  |
| Females |  |  |  |
| - Mortality age 0 to 4 months | 62 ± 6.2 |  | 62 ± 6.2 |
| - Mortality age 4 to 8 months | 18 ± 2.1 |  | 29 ± 4.8 |
| - Annual mortality > 8 months | 18 ± 2.1 |  | 29 ± 4.8 |
| Males |  |  |  |
| - Mortality age 0 to 4 months | 62 ± 6.2 |  | 62 ± 6.2 |
| - Mortality age 4 to 8 months | 32 ± 8.9 |  | 29 ± 4.2 |
| - Annual mortality > 8 months | 32 ± 8.9 |  | 29 ± 4.2 |
| **Catastrophes** |  |  |  |
| Fire (local/regional) |  |  |  |
| - Frequency % |  | 2.5^*4^ |  |
| Severity (proportion of normal values) |  |  |  |
| - Reproduction |  | 1 |  |
| - Survival |  | 0.3 |  |
| **Mate monopolization** |  |  |  |
| % males in breeding pool (relative mating success for young and older males) | = IF (A>=5; 60; 30) | | |
| **Initial population size** |  |  |  |
| Initial abundances | 37 (R1 = 23; R2 = 4;  R3 = 3; R4 = 7) |  | 27 (M1 = 9; M2 = 18) |
| (Stable age distribution) |  |  |  |
| **Carrying capacity** |  |  |  |
| Carrying capacity (*K*) | 85 (R1 = 23 ± 2.3; R2 = 4 ± 0.4; R3 = 19 ± 1.9; R4 = 39 ± 3.9) |  | 116 (M1 = 39 ± 3.9; M2 = 77 ± 7.7) |
| **Genetics** |  |  |  |
| Number of neutral loci to be modelled | 13 (12 real, 1 simulated) | | |
| Initial allele frequencies | S2 Table A |  | S2 Table A |
| Number of loci to be subject to mutation |  | 13 |  |
| - Mutation rate |  | 0.0001 |  |
| Genetic management |  |  |  |
| - Pair according to mean kinships | Use a dynamic MK list | | |

^*1^([5](#_ENREF_5)); ^*2^([6](#_ENREF_6)); ^*^([3](#_ENREF_3)); ^*4^Ramalho, unpublished data.

Paternity analysis (Ottewell and Chambers, unpublished data) informed several parameters in the Vortex model not available in RAMAS GIS, including male mate monopolization (paternity analysis showed 9/17 males gained paternity in Roe Highway, rounded to 60% male breeding success). Paternity analysis also showed that matings preferentially occurred between unrelated individuals and that inbreeding is currently low in the study populations (F = -0.02 and F = -0.01 for Mandjoogoordap Drive and Roe Highway metapopulations, respectively; unpublished data). In Vortex, we used the genetic management option to pair unrelated individuals using a dynamic MK list to reflect this mating behaviour.

Two levels of inbreeding depression were applied. Because the genetic load of *I. obesulus* populations is unknown, we firstly applied a ‘mild’ inbreeding depression rate of 3.14 diploid lethal equivalents, which is the median estimate from a meta-analysis of animals kept in captivity ([5](#_ENREF_5)). As inbreeding depression is stronger in stressful than benign environments ([7](#_ENREF_7)), we also applied a more realistic, ‘stressful’ level of inbreeding depression using the mean estimate of 6.29 diploid lethal equivalents on fecundity and first-year survival from a meta-analysis of wild populations ([6](#_ENREF_6)). Inbreeding depression was modelled as a reduction in survival of inbred juveniles.

To track genetic diversity change through time, the Vortex model was seeded with allele frequency data from 12 microsatellite loci genotyped in the Roe Highway and Mandjoogoordap Drive metapopulations (S2 Table A). Microsatellite and genotyping conditions were as described in ([8](#_ENREF_8)). We tracked changes in observed (H_O_) and expected heterozygosity (H_E_), the number of alleles, and population genetic differentiation (G_ST_) over the duration of each model. We calculated Wright’s inbreeding coefficient from observed and expected heterozygosity using F_IS_ = (H_E_ – H_O_)/H_E_. In some scenarios where extinction risks were high and population size fluctuated markedly, negative values of F_IS_ were observed due to the small sample sizes from which genetic diversity statistics were calculated. Similarly, in scenarios that resulted in ~100% decline in EMA (*e.g*., ‘stressful’ inbreeding depression) we were unable to calculate F_IS_ or G_ST_ due to small sample sizes.

## Literature cited

1. Akcakaya HR. RAMAS GIS: linking spatial data with population viability analysis. Version 5. Applied Biomathematics. Version 5 ed. Setauket, NY2005.

2. Lacy RC, Pollack JP. Vortex: A stochastic simulation of the extinction process. Version 10.0 ed. Brookfield, Illinois, USA: Chicago Zoological Society; 2014.

3. Southwell DM, Lechner AM, Coates T, Wintle BA. The sensitivity of population viability analysis to uncertainty about habitat requirements: implications for the management of the endangered southern brown bandicoot. Conservation Biology. 2008;22(4):1045-54.

4. Hone J, Duncan RP, Forsyth DM. Estimates of maximum annual population growth rates (rm) of mammals and their application in wildlife management. Journal of Applied Ecology. 2010;47(3):507-14.

5. Ralls K, Ballou JD, Templeton A. Estimates of lethal equivalents and the cost of inbreeding in mammals. Conservation Biology. 1988;2(2):185-93.

6. O’Grady JJ, Brook BW, Reed DH, Ballou JD, Tonkyn DW, Frankham R. Realistic levels of inbreeding depression strongly affect extinction risk in wild populations. Biological Conservation. 2006;133(1):42-51.

7. Armbruster P, Reed DH. Inbreeding depression in benign and stressful environments. Heredity. 2005;95(3):235-42.

8. Ottewell K, Dunlop J, Thomas N, Morris K, Coates D, Byrne M. Evaluating success of translocations in maintaining genetic diversity in a threatened mammal. Biological Conservation. 2014;171:209-19.
