# Supplementary material for: Demographic and genetic viability of a medium-sized ground-dwelling mammal in a fire prone, rapidly urbanizing landscape
Source: PLoS One. 2018 Feb 14;13(2):e0191190. doi: 10.1371/journal.pone.0191190 (PMC5812552; doi:10.1371/journal.pone.0191190)
Supplement: S1 Table — (DOCX) [file pone.0191190.s002.docx]

# S1 Table. Allele frequency and genetic diversity in the study metapopulations

Allele frequency data for 12 microsatellite loci genotyped in the Roe Highway (N=78) and Mandjoogoordap Drive (N=86) metapopulations.

| **Locus** | **Allele 1** | **Allele 2** | **Allele 3** | **Allele 4** | **Allele 5** | **Allele 6** | **Allele 7** | **Allele 8** | **Allele 9** |
| --- | --- | --- | --- | --- | --- | --- | --- | --- | --- |
| *Roe Highway* | | | | | | | | | |
| B7-2 | 0.09 | 0.071 | 0.167 | 0.179 | 0.269 | 0.128 | 0.096 |  |  |
| IOO8 | 0.071 | 0.205 | 0.026 | 0.179 | 0.301 | 0.218 |  |  |  |
| B34-2 | 0.596 | 0.019 | 0.013 | 0.051 | 0.115 | 0.006 | 0.199 |  |  |
| IOO6 | 0.084 | 0.039 | 0.721 | 0.032 | 0.123 |  |  |  |  |
| B20-5 | 0.092 | 0.079 | 0.257 | 0.007 | 0.132 | 0.013 | 0.066 | 0.316 | 0.039 |
| B3-2 | 0.227 | 0.318 | 0.039 | 0.279 | 0.091 | 0.026 | 0.006 | 0.013 |  |
| IOO10 | 0.336 | 0.013 | 0.303 | 0.013 | 0.336 |  |  |  |  |
| IOO4 | 0.244 | 0.006 | 0.16 | 0.301 | 0.019 | 0.09 | 0.179 |  |  |
| B34-1 | 0.359 | 0.199 | 0.103 | 0.045 | 0.295 |  |  |  |  |
| IOO16 | 0.25 | 0.378 | 0.372 |  |  |  |  |  |  |
| IOO2 | 0.013 | 0.679 | 0.301 | 0.006 |  |  |  |  |  |
| IOO7 | 0.072 | 0.316 | 0.237 | 0.362 | 0.013 |  |  |  |  |
| *Mandjoogoordap Drive* | | | | | | | | | |
| B7-2 | 0.194 | 0.271 | 0.059 | 0.018 | 0.094 | 0.153 | 0.212 |  |  |
| IOO8 | 0.271 | 0.424 | 0.018 | 0.135 | 0.141 | 0.012 |  |  |  |
| B34-2 | 0.345 | 0.542 | 0.012 | 0.077 | 0.024 |  |  |  |  |
| IOO6 | 0.494 | 0.024 | 0.282 | 0.194 | 0.006 |  |  |  |  |
| B20-5 | 0.247 | 0.018 | 0.006 | 0.112 | 0.047 | 0.012 | 0.312 | 0.247 |  |
| B3-2 | 0.4 | 0.006 | 0.265 | 0.182 | 0.035 | 0.041 | 0.071 |  |  |
| IOO10 | 0.006 | 0.276 | 0.253 | 0.435 | 0.006 | 0.024 |  |  |  |
| IOO4 | 0.012 | 0.171 | 0.265 | 0.082 | 0.047 | 0.424 |  |  |  |
| B34-1 | 0.041 | 0.035 | 0.159 | 0.218 | 0.006 | 0.482 | 0.059 |  |  |
| IOO16 | 0.129 | 0.618 | 0.041 | 0.212 |  |  |  |  |  |
| IOO2 | 0.306 | 0.688 | 0.006 |  |  |  |  |  |  |
| IOO7 | 0.006 | 0.035 | 0.147 | 0.053 | 0.276 | 0.182 | 0.088 | 0.106 | 0.106 |

**Table B.** Mean and standard error of initial genetic diversity parameters (*i.e*., at year 0) for *I. obesulus* metapopulations at Roe Highway and Mandjoogoordap Drive. N = mean metapopulation size; N_a_ = mean number of alleles per locus; H_e_ = mean expected heterozygosity; H_o_ = mean observed heterozygosity; and F_IS_ = mean inbreeding coefficient.

| **Metapopulation** | **N** | **N_a_** | **H_e_** | **H_o_** | **F_IS_** |
| --- | --- | --- | --- | --- | --- |
| Roe Highway | 37 ± 0.0 | 5.35 ± 0.01 | 0.675 ± 0.0003 | 0.684 ± 0.0007 | -0.013 |
| Mandjoogoordap Drive | 27 ± 0.0 | 5.18 ± 0.01 | 0.664 ± 0.0003 | 0.676 ± 0.0008 | -0.017 |
