# Supplementary material for: Demographic and genetic viability of a medium-sized ground-dwelling mammal in a fire prone, rapidly urbanizing landscape
Source: PLoS One. 2018 Feb 14;13(2):e0191190. doi: 10.1371/journal.pone.0191190 (PMC5812552; doi:10.1371/journal.pone.0191190)
Supplement: S1 Fig — (DOCX) [file pone.0191190.s003.docx]

# S1 Fig. Expected minimum abundance under different management scenarios estimated using RAMAS and Vortex software

Fig. Expected minimum abundance (EMA) for the Roe Highway (black markers) and Mandjoogoordap Drive (white markers) metapopulations under the different management scenarios, using RAMAS GIS (solid lines) and Vortex (dashed lines) software.
