# Supplementary material for: Demographic and genetic viability of a medium-sized ground-dwelling mammal in a fire prone, rapidly urbanizing landscape
Source: PLoS One. 2018 Feb 14;13(2):e0191190. doi: 10.1371/journal.pone.0191190 (PMC5812552; doi:10.1371/journal.pone.0191190)
Supplement: S2 Fig — (DOCX) [file pone.0191190.s004.docx]

# S2 Fig. Additional results

(a)

(b)

**Fig A.** Trajectory of the base model, in terms of estimated average population abundance, for the (a) Roe Highway and (b) Mandjoogoordap Drive metapopulations (standard deviation represented with grey lines).

**Fig B.** Percent decline in expected minimum abundance (EMA) in relation to the base model for the Roe Highway (solid line) and Mandjoogoordap Drive (dashed line) metapopulations, under the main management scenarios impacting on dispersal (underpasses and urbanization), with and without considering inbreeding depression. Two rates of inbreeding depression were considered: mild (mID) and stressful (sID).

**Fig C.** Genetic differentiation (G_ST_) amongst habitat patches for different management scenarios after 50 years, for the Roe Highway (solid line) and Mandjoogoordap Drive (dashed line) metapopulations. G_ST_ was not calculated for scenarios with high extinction probabilities due to small sample size.
